# Supplementary material for: Expression, Characterization and Selective Chemical Inhibition of Essential Schistosoma mansoni Tegumental Acetylcholinesterase (SmTAChE)
Source: Int J Mol Sci. 2025 Feb 25;26(5):1975. doi: 10.3390/ijms26051975 (PMC11900278; doi:10.3390/ijms26051975)
Supplement: Supplementary file 1 [file ijms-26-01975-s001.zip › Supplemenray Figures.pdf]

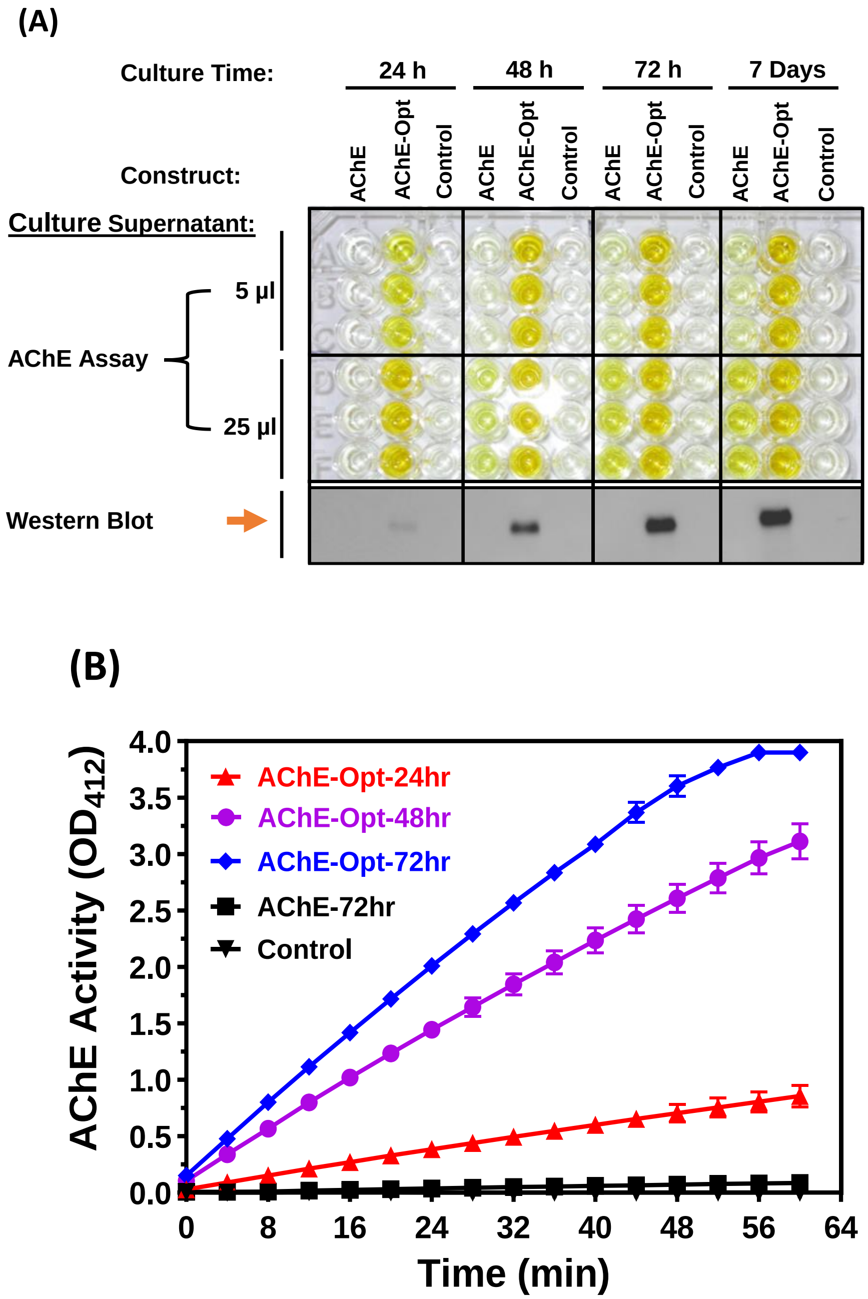

Supplementary Figure S1. Codon optimization greatly improves rSmTAChE production in CHO-S cells. CHO-S cells were transfected with plasmid encoding standard (i.e., non-codon-optimized) SmTAChE (AChE), or hamster codon-optimized SmTAChE (AChE-Opt), or with empty plasmid (control). Supernatants were analyzed for AChE activity using 5µl or 25µl aliquots of culture medium, as noted. **(A)** Enzyme assay plate showing the activity of rSmTAChE (in triplicate, after 1h incubation) in culture supernatants collected from cells transfected with the indicated plasmids (at 24h, 48h, 72h or 7 days after transfection, as indicated at top). Yellow color indicates activity of rSmTAChE. Supernatant aliquots were resolved by SDS-PAGE and analyzed by western blotting using anti-SmTAChE antibody (**A, bottom panel**). The orange arrow points to rSmTAChE. **(B)** Activity plots of rSmTAChE (OD_{412} ± SD) over time in 5 µl of culture supernatant collected and tested as described in the text, in triplicate and at the indicated time points.

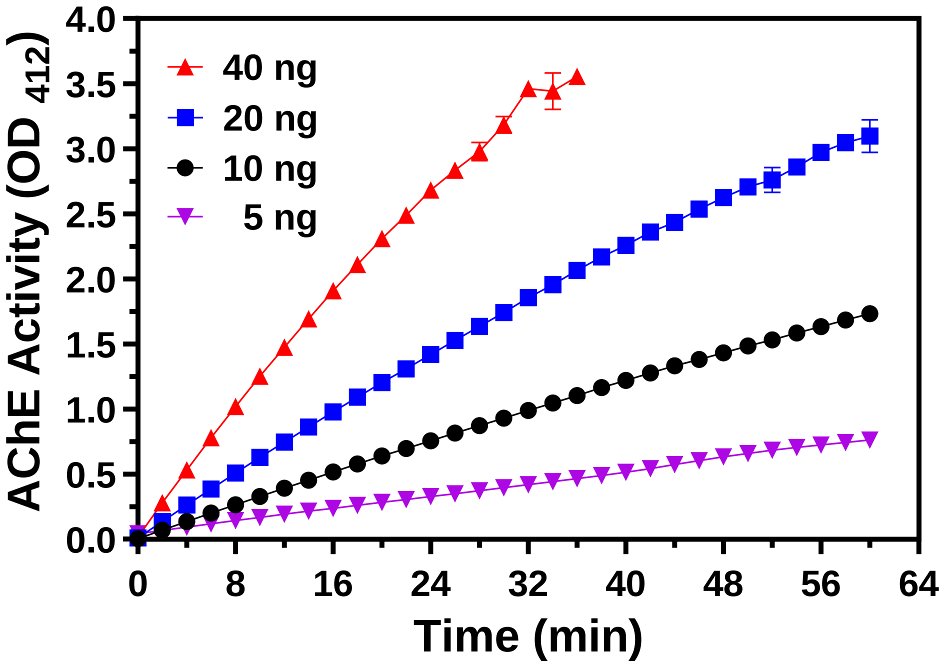

Supplementary Figure S2. The effect of rSmTAChE concentration on ATCh hydrolysis. Activity plots (OD_{412} ± SD) of different amounts of purified rSmTAChE (5ng – 40ng, as indicated, in triplicate) each showing good linearity profiles in the time frames examined.

atgctgctgtcctttacccatctgtccgtcgtgagcctgttttctatcatcatcattccaactactgtgaagtca
M L L S F T H L S V V S L F S I I I I P T T V K S

aat**acc**ctgaagcctatcgaaattccactgacccacggcggctccgtgatcggaaaagaggaaatcgtcaacatt
N **T** L K P I E I P L T H G G S V I G K E E I V N I

gacgggcaggaggtgaaggtcaatagtttcctgggtattccatacgcatcaaagcccatcggcaaactgagattt
D G Q E V K V N S F L G I P Y A S K P I G K L R F

gcccctccagagaagcatcctggatggaaggggaagtataacgcaaccacactgagcccaacatgctggcagtac
A P P E K H P G W K G K Y N A T T L S P T C W Q Y

attttcactggttttgatgccgtgaatgccgctggcaaaatgtggatcaacaataccgagatgagcgaagactgt
I F T G F D A V N A A G K M W I N N T E M S E D C

ctgtatctgaacgtgtggacaccaaagtccagcgtcgattctccccacctgcctgtgatggtctggatctacggg
L Y L N V W T P K S S V D S P H L P V M V W I Y G

ggtggcttcacaagcggatctgctaacctgcaggtgtacaatggggccatcctgtccgctactcagaacgtgatc
G G F T S G S A N L Q V Y N G A I L S A T Q N V I

attgtcagcatgcagtacagagtgggtgcattcggctttctgcgcctgaaacccaacatcactgaccagactcag
I V S M Q Y R V G A F G F L R L K P N I T D Q T Q

accgatgctctgggaaatcaggggctgctggaccagctgatggcactgaagtgggtgagcgaaaacattggccag
T D A L G N Q G L L D Q L M A L K W V S E N I G Q

ttccacggcgatcctaatcaggtcaccatctttggagagtcagcaggggccgtgagtgtctcaattctgtggatg
F H G D P N Q V T I F G E S A G A V S V S I L W M

tcccccatcgcccagccttatttcaggcgggctatcctgcagtccggaagcctgtacgcacgatggggctggac
S P I A Q P Y F R R A I L Q S G S L Y A R W G L D

aacgctgatgaggcacatgaaaaggctgacgtgtttacccgtgaatgcggatgtcagtctcccagtgtcgatcga
N A D E A H E K A D V F T R E C G C Q S P S V D R

aaggccagtctggagtgcctgcgtaaactggaccctctgacactggtgaaccagctggattcaattaatgtcgct
K A S L E C L R K L D P L T L V N Q L D S I N V A

atcggcaagcacagatatgacgcagtgcggaaatatctgctgccaagataccataagcaggagcccttcctgctg
I G K H R Y D A V R K Y L L P R Y H K Q E P F L L

tcacagtccacaagcactcgcctgtattttgacgtgccactgcagcccgtcattgatggctacctggtgcctaag
S Q S T S T R L Y F D V P L Q P V I D G Y L V P K

cacccagaccatatcttcaacgagaaaaataagctgaaacagaatccagaactgctgatcggtgtgaacaccaat
H P D H I F N E K N K L K Q N P E L L I G V N T N

gaggccatgttctttctgctgcccggcatcgccatcaaggatactcagttcctgttttcaaacggctccgtgatc
E A M F F L L P G I A I K D T Q F L F S N G S V I

atgccctccacaatggagctggccggaaagaaaaagcctttcaaggaggggggaggaaatcgcagacttttactgg
M P S T M E L A G K K K P F K E G E E I A D F Y W

attaccgccacacagatcctggatgagtctcacatgcgccctgggctggctaaaatgccaagttactattacaat
I T A T Q I L D E S H M R P G L A K M P S Y Y Y N

ctgccacccacatctagtcctaagcgaggctattacgacccagataccgtgtacatccatgacgaggaactgctg
L P P T S S P K R G Y Y D P D T V Y I H D E E L L

agacgcctggataagttcgctggcgacctggattttgcatgtccaaccctgaactttgcagaacaggtggccagg
R R L D K F A G D L D F A C P T L N F A E Q V A R

ctgcccaacgctaaagtcttcctgtaccactttaataagcggaccgagagtctgcctatgccaaagtggacaggt
L P N A K V F L Y H F N K R T E S L P M P K W T G

gtgatgcatggctatgagatcgaatacattttcggaatcccctatgaccctgaattttccaagcagttctacaac
V M H G Y E I E Y I F G I P Y D P E F S K Q F Y N

ttcacagatcctgagaagattttctcatccaggatcatgaagatgtggactaactttgccaaaaccggtcaccca
F T D P E K I F S S R I M K M W T N F A K T G H P

tctaagagtaatgacggcaaaatttccactccagagtggcccctgttccatagcaccgatggcttcgtgtccaac
S K S N D G K I S T P E W P L F H S T D G F V S N

aatcctgactacctgatcctggaggatgaaacaaagctgggtagcggactgcaccgagaccgttgcgctttctgg
N P D Y L I L E D E T K L G S G L H R D R C A F W

ctgcatgagatgcaggacatgaaagatatt**tgg**tttaaccggtgtgatccctctggagggatcaagcctactggc
L H E M Q D M K D I **W** F N R C D P S G G I K P T G

aactacatcctgattctggggagcggtctgctgctgtttattggcatcttttatgggtga
N Y I L I L G S G L L L F I G I F Y G *

Supplementary Figure S3. The codon-optimized nucleotide sequence of SmTAChE. The corresponding amino acid sequence is displayed below the nucleotide sequence. The region coding for amino acids T^{27} to W^{661} (marked in red and highlighted in yellow) was amplified by PCR and cloned into the pSecTag2A plasmid, as described under the Materials and Methods section.
