# Supplementary material for: Expression, Characterization and Selective Chemical Inhibition of Essential Schistosoma mansoni Tegumental Acetylcholinesterase (SmTAChE)
Source: Int J Mol Sci. 2025 Feb 25;26(5):1975. doi: 10.3390/ijms26051975 (PMC11900278; doi:10.3390/ijms26051975)
Supplement: Supplementary file 1 [file ijms-26-01975-s001.zip › Supplemenray Tables.pdf]

Supplemental Table S1. Structure and activity of compound's #2 synthetic analogs.

No.	Compound ID	Structure	Formula Weight	IC50 (µM)	
**1**	Cpd.1	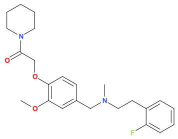	414.5	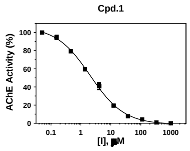	2.0
**2**	Cpd.2	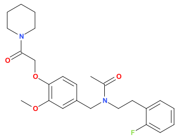	442.5	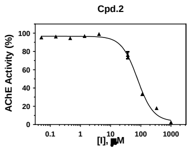	77.4
**3**	Cpd.3	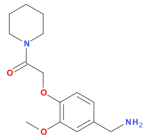	278.4	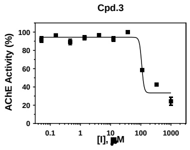	>100.0
**4**	Cpd.4	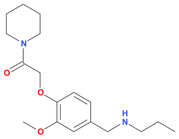	320.4	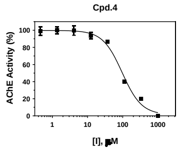	96.3
**5**	Cpd.5	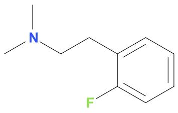	167.2	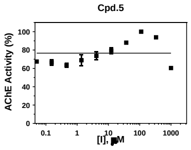	ND
**6**	Cpd.6	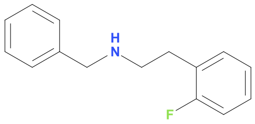	229.3	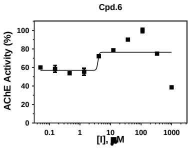	ND
**7**	Cpd.7	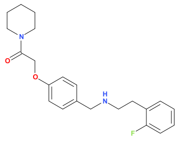	370.5	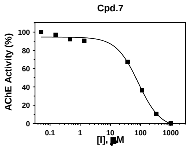	83.8
**8**	Cpd.8	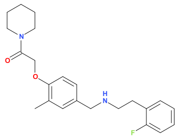	384.5	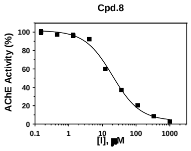	20.0
**9**	Cpd.9	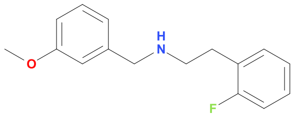	259.3	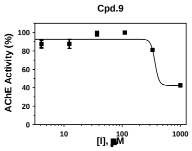	>367
**10**	Cpd.10	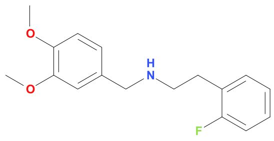	289.3	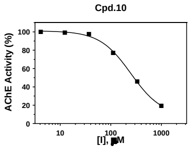	294.6

**11**	Cpd.11	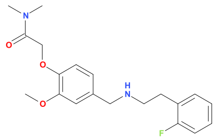	360.4	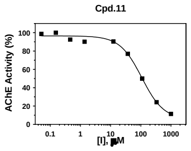	107.9
**12**	Cpd.12	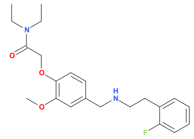	388.5	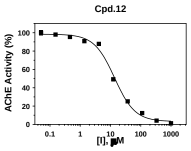	13.6
**13**	Cpd.13	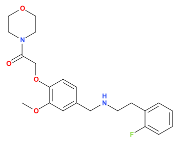	402.5	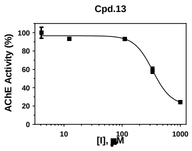	335
**14**	Cpd.14	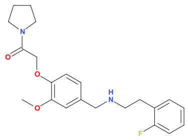	386.5	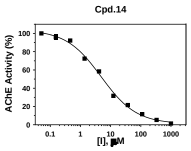	4.88
**15**	Cpd.15	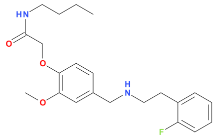	388.5	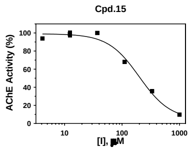	199.3
**16**	Cpd.16	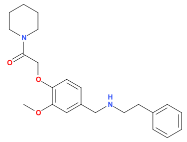	382.5	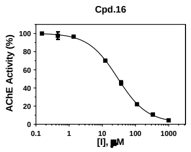	29.0
**17**	Cpd.17	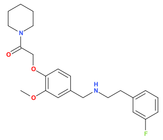	400.5	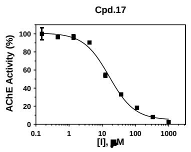	16.2
**18**	Cpd.18	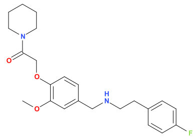	400.5	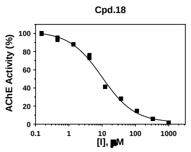	9.9
**19**	Cpd.19	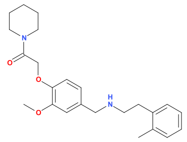	396.5	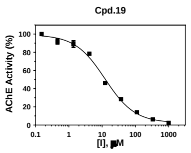	12.2
**20**	Cpd.20	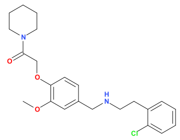	416.9	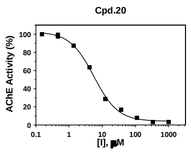	5.6
**21**	Cpd.21	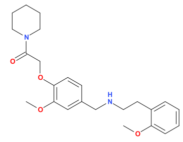	412.5	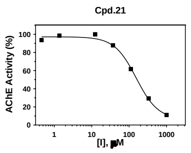	154.7

**22**	Cpd.22	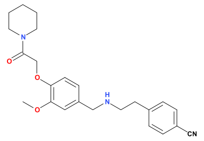	407.5	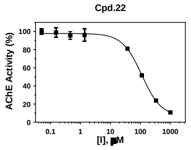	111.6
**23**	Cpd.23	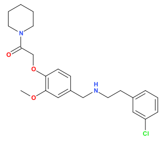	416.9	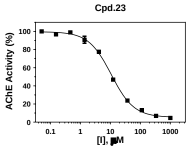	10.6
**24**	Cpd.24	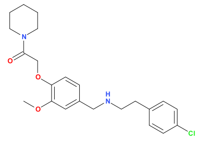	416.9	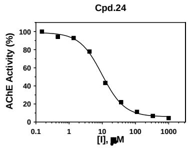	9.8
**25**	Cpd.25	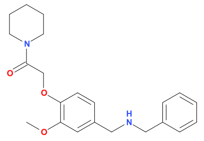	368.5	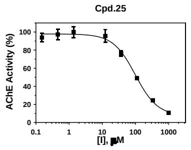	99.4

ND: Not Determined
